# Clinical significance of T cell metabolic reprogramming in cancer

**DOI:** 10.1186/s40169-016-0110-9

**Published:** 2016-08-10

**Authors:** Christoph Herbel, Nikolaos Patsoukis, Kankana Bardhan, Pankaj Seth, Jessica D. Weaver, Vassiliki A. Boussiotis

**Affiliations:** 1 Division of Hematology-Oncology, Department of Medicine, Beth Israel Deaconess Medical Center, Harvard Medical School, Boston, MA 02215 USA; 2 Beth Israel Deaconess Cancer Center, Harvard Medical School, 330 Brookline Avenue, Dana 513, Boston, MA 02215 USA; 3 Division of Interdisciplinary Medicine and Biotechnology, Beth Israel Deaconess Medical Center, Boston, USA

## Abstract

Conversion of normal cells to cancer is accompanied with changes in their metabolism. During this conversion, cell metabolism undergoes a shift from oxidative phosphorylation to aerobic glycolysis, also known as Warburg effect, which is a hallmark for cancer cell metabolism. In cancer cells, glycolysis functions in parallel with the TCA cycle and other metabolic pathways to enhance biosynthetic processes and thus support proliferation and growth. Similar metabolic features are observed in T cells during activation but, in contrast to cancer, metabolic transitions in T cells are part of a physiological process. Currently, there is intense interest in understanding the cause and effect relationship between metabolic reprogramming and T cell differentiation. After the recent success of cancer immunotherapy, the crosstalk between immune system and cancer has come to the forefront of clinical and basic research. One of the key goals is to delineate how metabolic alterations of cancer influence metabolism-regulated function and differentiation of tumor resident T cells and how such effects might be altered by immunotherapy. Here, we review the unique metabolic features of cancer, the implications of cancer metabolism on T cell metabolic reprogramming during antigen encounters, and the translational prospective of harnessing metabolism in cancer and T cells for cancer therapy.

## Cancer cell metabolism and implications on T cell function in the tumor microenvironment

Since the early days of cancer biology research, it was determined that cancer cells acquire novel metabolic properties [1]. In a seminal discovery in 1923, Otto Warburg identified that cancer cells are characterized by an irreversible transition of their energy-producing machinery from mitochondrial respiration, where oxidative phosphorylation (OXPHOS) occurs, to glycolysis, a biochemical process that occurs in the cytoplasm without oxygen requirement, which can occur under aerobic and hypoxic conditions. Glycolysis results in the production of ATP and lactate and is the preferred metabolic program of cancer cells even in presence of sufficient amounts of oxygen that could support OXPHOS. However, it was later appreciated that tumor cells also utilize OXPHOS [2–5] and that depletion of mitochondrial function mainly compromises the stemness features of cancer [6]. The very small percentage of this OXPHOS-dependent fraction of cancer cells within the predominantly glycolytic cell population in tumors was the reason for which the role of OXPHOS in cancer remained unnoticed and neglected.

In addition to being the predominant metabolic program of growing cancer cells, aerobic glycolysis is also operative during physiological states in the life of T cells. Naïve T cells utilize OXPHOS for energy generation, but upon activation via the T cell receptor (TCR), switch their metabolic program to glycolysis. Although energetically less efficient due to the production of lower number of ATP molecules per molecule of glucose compared to OXPHOS, glycolysis is required to support T cell effector differentiation and function [7, 8]. Various experimental findings support the hypothesis that glycolysis has a selective advantage over oxidative phosphorylation during T cell activation. Glycolysis has higher ATP generation rate, can function under hypoxic and/or acidic conditions, and provides higher biosynthetic benefit and better maintenance of redox balance than OXPHOS [9]. These properties of glycolysis are also beneficial for cancer cells [10]. However, an important difference between glycolysis in activated T cells and cancer cells is that, in cancer cells, this metabolic program is a consequence of cellular dysregulation due to oncogenic mutations, while in T cells glycolysis represents a physiologically regulated metabolic adaptation [9, 11]. During exposure to activating external queues such as antigen, costimulatory signals, and cytokines, T cells also upregulate inhibitory receptors, which oppose the effects of activation signals and provide regulation of immune homeostasis and prevention of autoimmunity. Importantly, tumors evade the immune system by expressing specific ligands for these inhibitory receptors, prototyped by PD-1, thus causing and maintaining T cell immunosuppression [12, 13]. Via T cell intrinsic mechanisms, these inhibitory receptors directly oppose the physiologic metabolic reprogramming that occurs during T cell activation [14, 15].

A key mechanism by which cancer alters the functional fate of T cells is related to altered nutrient availability and metabolic state in the tumor microenvironment. Specifically, cancer cells develop glucose addiction and depend on glycolysis as their main metabolic program and thus acquire a high rate of glucose intake. As a consequence, T cells in the tumor microenrvironment undergo glucose deprivation due to high competition for glucose intake by cancer and activated T cells [16, 17]. In T lymphocytes, glucose uptake and catabolism is not simply a metabolic process for nutrient utilization and energy generation. Glycolysis has a key role on the T cell fate upon antigen-encounter and is mandatory for the differentiation of the naïve T cells into antigen-specific T effectors (T_EFF_) [7, 18, 19]. Thus, by creating a microenvironmental condition of glucose starvation for T cells, cancer inhibits the differentiation and expansion of tumor-specific T cells exposed to tumor associated antigens (TAA) that renders them unable to develop into tumor-specific T_EFF_ cells [17]. Instead, these metabolic conditions, promote differentiation of T cells into Treg [18]. In addition to glucose, an equally important metabolite required for T cell differentiation and function is glutamine. Sufficient supply of glutamine and its utilization by T cells has an indispensable role for the development of T_EFF_ cell fitness [20, 21]. Several cancers develop enhanced glutamine metabolism as a consequence of cell intrinsic carcinogenic events including mutations and altered signaling pathways thereby becoming glutamine addicted [22]. As a consequence tumor-specific T cells residing in the cancer microenvironment are subjected to glutamine deprivation in addition to glucose competition.

Cancer cells not only compete for availability of key nutrients required for T cell activation, T_EFF_ differentiation and adaptation of anti-tumor fitness, but also produce metabolic products, which are harmful for T cells [23]. To this end, it is important to understand the unique metabolic features of cancer and their implications in the tumor microenvironment and subsequently to resident T cells. Mechanistic understanding of these metabolic balances will provide the means to develop novel strategies for therapeutic targeting in order to harness the maximum anti-tumor potential of the adaptive immune system. A stepwise description of these cancer-specific metabolic modulations is outlined in the following sections.

## Metabolic features of cancer cells: glycolysis and Warburg effect, metabolic flexibility, metabolic flux

### Adaptation to glycolysis and the Warburg effect

**Fig. 1:**
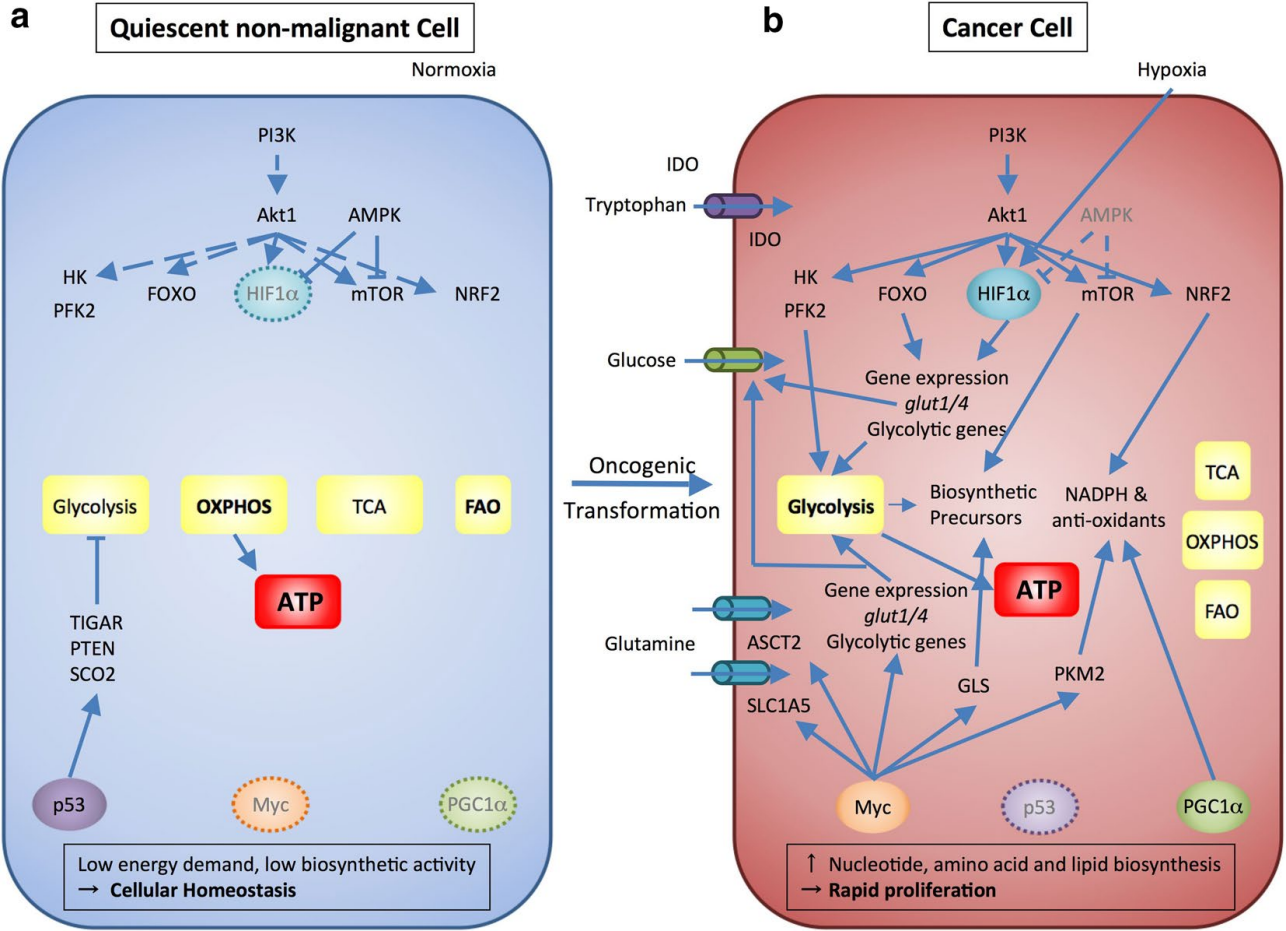
Molecular alterations cause metabolic switching in cancer cells and severe metabolic changes in the tumor microenvironment. **a** Non malignant (quiescent) cells rely on OXPHOS as primary ATP source under normoxic conditions. FAO also contributes to the cellular ATP pool. Without extrinsic stimuli the PI3K-Akt1 pathway is inactive. Downstream targets are also blocked, e.g. HK, PFK2, FOXO, HIF1α, mTOR, and NRF2. In addition, AMPK keeps HIF1α and mTOR in check. p53 participates in the repression of glycolysis by expression of TIGAR, PTEN, and SCO2. Myc as well as PGC1α are not active in quiescent cells and do not contribute to glycolysis. In order to sustain cellular homeostasis cells have a low energy demand and low biosynthetic activity. **b** Cancer cells acquire a series of mutations that foster glycolysis in several ways. Oncogenic PI3K-Akt1 signaling and inhibited AMPK signaling promote activation of pro-glycolytic events. These include activation of glycolytic enzymes namely HK and PFK2 and activation of transcription factors such as FOXO and in combination with hypoxia- HIF1α, which in turn induce the expression of glucose transporters glut1 and glut4 and other glycolytic enzymes. Moreover, mTOR signaling is elevated which causes an increase in biosynthetic precursors. PI3K-Akt1-activated NRF2 induces expression of glycolytic genes as well as NADPH and anti-oxidants. PGC1α can also contribute to the cellular anti-oxidant pool. Mutation or deletion of p53 causes loss of glycolytic inhibitors like TIGAR, PTEN, and SCO2. Oncogenic Myc induces the expression of glycolytic genes, glucose and glutamine transporters. Additionally, Myc enhances the amount of biosynthetic precursors by expression of GLS and the amount of cellular NAPDH and anti-oxidants via PKM2. Expression of IDO mediates the degradation of tryptophan to *N*-formylkynurenin, the first step of tryptophan catabolism in the kynurenin pathway. These mutations elevate nucleotide, amino acid, and lipid biosynthesis paired with enhanced catabolic pathways to enable cancer cells to proliferate rapidly

During oncogenesis, cancer cells acquire several features, which discriminate them from their nonmalignant counterparts. One of these features is a change in the cellular metabolic program [10]. Cancer cells undergo metabolic adaptation and thereby gain selective survival and growth advantage (Fig. 1). Under physiological conditions most nonmalignant cells rely on OXPHOS as a primary metabolic pathway to generate energy in the form of adenosine triphosphate (ATP). Cancer cells, however, switch to glycolysis as primary energy source even in the presence of sufficient amounts of oxygen to support OXPHOS, a phenomenon known as the “Warburg effect” [24]. Nevertheless, it should be pointed out that cancer cells have a significant degree of metabolic flexibility and rely on fatty acid β-oxidation (FAO) and OXPHOS for their needs, but these metabolic pathways are not primary sources of ATP. OXPHOS utilizes glucose-derived pyruvate produced through glycolysis, and oxygen in the mitochondria to generate ATP, CO_2_, and H_2_O. Aerobic glycolysis, in contrast, generates ATP and lactate in the cytosol from glucose-derived pyruvate. Although aerobic glycolysis per se is less efficient in terms of ATP generation (glycolysis produces 2 mol of ATP per mole of glucose, whereas mitochondrial respiration generates 36 mol of ATP per mole of glucose [25]), cancer cells circumvent this drawback by increasing the rate of glycolysis. Moreover, they benefit from other aspects of metabolism induced by aerobic glycolysis, such as elevated amounts of biosynthetic precursors and increased reducing potential, in the form of NADH that is generated from the glycolytic pathway and in the form of NADPH that is generated from the pentose phosphate pathway (PPP), to which glycolytic mediators are shunted. Thus, the Warburg effect influences not only anabolic pathways leading to selective growth advantage of cancer cells but also supports redox balance and generation of biosynthetic precursors. Because glycolysis can operate either in the presence or in the absence of oxygen, adaptation of the Warburg effect ensures that cancer cells can use the same metabolic program, i.e. glycolytic, under aerobic as well as anaerobic/hypoxic conditions, which confers metabolic stability of cancer cells.

The molecular drivers causing the switch of cancer cell metabolism to aerobic glycolysis are diverse and are likely to act synergistically. Extensive studies have provided evidence that a series of sequential and independent mutations are required to develop the full potential of the Warburg effect [10, 24, 26]. Certain signaling pathways involved by such mutations or gene amplifications, outlined below, have key roles in regulating mechanisms that lead to the adaptation of the Warburg effect as the metabolic hallmark of cancer cells.

#### PI3K-Akt-mTOR-FOXO

The phosphoinositide 3-kinase (PI3K) pathway is one of the most frequently altered signaling pathways in human cancers. Under physiological conditions the PI3Ks are a family of proteins involved in the regulation of cell survival, growth, metabolism, and glucose homeostasis [27]. This pathway is activated by mutations in tumor suppressor genes, such as phosphatase and tensin homolog (PTEN), mutations in the components of the PI3K complex, or by elevated signaling from receptor tyrosine kinases [28]. Upon activation, the PI3K pathway not only provides strong growth and survival signals to tumor cells but also has strong effects on their metabolism. The best-studied effector downstream of PI3K is the RAC-alpha serine/threonine-protein kinase (AKT1). AKT1 is a critical driver of the tumor glycolytic phenotype and stimulates ATP generation through multiple mechanisms, ensuring that cells have the bioenergetic capacity required to respond to growth signals [29, 30]. AKT1 stimulates the expression of glucose transporters, phosphorylates key glycolytic enzymes, and alters their catalytic activity, e.g. hexokinase (HK) and phosphofructokinase 2 [29, 31]. In addition, the activation and the prolonged signaling via AKT1 inhibits forkhead box subfamily O (FOXO) transcription factors by mediating their phosphorylation and sequestration in the cytoplasm, resulting in transcriptional changes that increase glycolytic capacity [32]. AKT1 also directly influences the mammalian target of rapamycin (mTOR) signaling pathway. mTOR functions as a key metabolic hub, coupling growth signals to nutrient availability. Under physiological stimulation triggered by amino acids, growth factors, stress, energy, as well as oxygen, mTOR stimulates protein and lipid biosynthesis and cell growth when sufficient nutrient and energy are available [33–35]. mTOR is often constitutively activated during tumorigenesis [36]. AKT1 strongly activates mTOR-signaling pathway by inducing an inhibitory phosphorylation of tuberous sclerosis 2 (TSC2), a negative regulator of mTOR [27, 31].

#### AMPK-mTOR

AMP-activated protein kinase (AMPK) is a crucial sensor of energy status and has an important role in cellular responses to metabolic stress. Like the mTOR pathway, the AMPK pathway couples energy status to growth signals. However, AMPK opposes the effects of AKT1 and functions as an inhibitor of mTOR. AMPK is activated by an increased AMP/ATP ratio and is responsible for shifting cells to OXPHOS and inhibiting cell proliferation [37]. AMPK inhibits the activation of mTOR complex indirectly by mediating phosphorylation of the mTOR upstream regulator TSC2 to keep it suppressed [38] or directly via phosphorylation of the mTOR regulatory component, Raptor, to trigger its sequestration by 14-3-3 and subsequent dissociation from mTOR [39]. In many types of cancer the liver kinase B1 (LKB1), the upstream activator for AMPK, is inactivated. This results in diminished AMPK signaling and loss of mTOR inhibition [37] and might support the shift of cancer cell metabolism towards glycolysis. Furthermore, loss of AMPK signaling mediates increased expression of hypoxia induced factor 1α (HIF1α) [40].

Deregulation of the mTOR pathway is often observed in human cancers, which is consistent with its critical role in regulating cell growth and metabolism [36, 41]. The most prominent function of mTOR is to control protein synthesis through directly phosphorylating translational regulators such as eukaryotic translation initiation factor 4E binding protein 1 (4E-BP1) and S6 kinase 1 (S6K1) [42]. By this mechanism, oncogenic mTOR triggers protein synthesis and enhances cell growth and proliferation. Consistently, mutations or loss-of-function of upstream regulatory genes, such as tuberous sclerosis complex 1/2 (TSC1/2), have been linked to cancer initiation and development [43].

#### p53-Myc

Although the transcription factor and tumor suppressor p53 is best known for its functions in the DNA damage response and apoptosis [44], it has become evident that p53 is also an important regulator of metabolism [45]. p53 inhibits the glycolytic pathway by upregulating the expression of TP53-induced glycolysis and apoptosis regulator (TIGAR), an enzyme that decreases the levels of the glycolytic activator fructose-2,6-bisphosphate [46]. Moreover, p53 supports the expression of PTEN, which inhibits the PI3K pathway, and thus suppresses glycolysis [47]. In addition, p53 promotes OXPHOS by fostering expression of cytochrome C oxidase assembly protein (SCO2), which is required for the assembly of the cytochrome C oxidase complex of the electron transport chain [48]. Consequently, besides its anti-apoptotic effect, the loss of p53 can be a major force behind the acquisition of the glycolytic phenotype of cancer cells [49, 50].

The transcription factor c-Myc has important metabolic roles in enhancing glycolysis, mitochondrial gene expression, and mitochondrial biogenesis [51]. Tumors overexpressing Myc also have increased metabolic flux [52]. Myc enhances transcription of genes encoding glucose transporters to increase glucose import and glycolytic genes including HK, phosphoglucose isomerase, phosphofructokinase, glyceraldehyde-3-phosphate dehydrogenase, phosphoglycerate kinase, and enolase [53]. Myc also promotes nucleotide and amino acid synthesis, both through direct transcriptional regulation and through increasing the synthesis of mitochondrial metabolite precursors [54]. The effects of Myc or oncogenic PI3K signaling can be further pronounced by the simultaneous action of the two pathways [55]. Since Myc and mTOR are master regulators of protein synthesis, one could anticipate that Myc coordinates with mTOR. Indeed, Myc inhibits the mTOR repressor TSC2, thereby increasing mTOR activity to facilitate translation through S6K and 4E-BP phosphorylation [56]. mTOR can also increase glutamine flux through S6K1-dependent c-Myc upregulation, which in turn increases glutaminase (GLS) activity [57].

#### Metabolic flux

The basic caveat of low efficiency-ATP-production through glycolysis is compensated by increase in metabolic flux. The uptake of glucose in cancer cells is elevated by increased expression of glucose transporters, e.g. *Glut1* [58] and *Glut4* [59]. Furthermore, glycolytic genes are up-regulated (e.g. HK) [53], amplified (e.g. HK2) [60], or alternatively spliced (e.g. PKM2) [61]. The latter two alterations preferentially impact the enzymatic activity of key enzymes. Thereby, cancer cells accelerate the glycolytic flux and compensate for the reduced efficacy of aerobic glycolysis. Consequently, the net amount of ATP generated by aerobic glycolysis is even higher compared to OXPHOS.

Additionally, cancer cells benefit from aerobic glycolysis and altered glycolytic-enzymatic activities by an increased production of biosynthetic precursors. After the first reaction of glycolysis by HK2, glucose is phosphorylated to glucose-6-phosphate (G6P). A proportion of G6P can be redirected into the pentosphosphate pathway (PPP) to generate NADPH and ribose 5-phosphate (R5P) [54]. NADPH itself is utilized for macromolecular biosynthesis and redox regulation [62]. R5P is also needed for nucleotide synthesis [62]. G6P may also be used in the hexosamine pathway, which provides the cell with Uridine diphosphate N-acetylglucosamine (UDP-GlcNAc), a co-substrate for protein glycosylation [63]. Another glycolysis product, 3-phosphoglycerate, feeds into the serine/glycine synthesis pathway fostering nucleotide and protein biosynthesis [64, 65].

#### Beyond Warburg

Metabolic adaptation of cancer cells extends beyond ATP production. Energy production is only one part of the growth equation. In parallel to energy generation, cancer cells require biosynthetic precursors in order to support their growth. Furthermore, because cancer cells generate reactive oxygen species (ROS) as a consequence of rapid proliferation, activation of mechanisms to sustain the balance of the intracellular redox level is a key component of metabolic adaptation. To meet their demands for biosynthetic precursors and to minimize metabolic damage, cancer cells have acquired unique biochemical properties. These alterations support survival and growth programs, adaptation to various microenvironmental conditions with minimum damage, and survival under stress and limited nutrient availability. To achieve these properties, cancer cells adopt molecular and biochemical programs, which facilitate nutrient utilization in a manner distinct from their normal counterparts. Such changes have major impact not only on cancer cells themselves by supporting their growth, but also generate metabolic products which alter the microenvironment and affect the fate and function of immune cells residing in the microenvironment of cancer.

#### Tryptophan

Tryptophan is an essential amino acid and has a key role in cell survival. The enzymes that initiate the first and rate-limiting step of tryptophan degradation to *N*-formylkynurenine during tryptophan catabolism in the kynurenine pathway are tryptophan 2,3-dioxygenase (TDO), indoleamine 2,3-dioxygenase 1 and 2 (IDO1 and IDO2, respectively) [66]. Although these enzymes catalyze the same biochemical reaction, they share limited structural similarity [67]. It is noteworthy that tumor cells do not upregulate expression of TDO but specifically overexpress IDO, which recognizes a broader range of substrates, including l- or d-tryptophan, serotonin, and tryptamine [68]. This allows cancer cells to utilize a wider range of amino acids by altering the expression of a single gene. At the end of the kynurenine pathway quinolinic acid is generated [69]. In a subsequent step, NAD^+^, an essential cofactor for cellular homeostasis, can be produced from quinolinic acid [70–73]. Thus, IDO overexpression in tumor cells promotes de novo NAD^+^ synthesis. The underlying mechanism and signals causing IDO overexpression in cancer cells are currently under intense investigation. Although the precise mechanism(s) remain uncertain, there is preliminary evidence that IFN-γ might be contributing to IDO expression in tumors [74].

#### Glutamine

Elevated glutamine consumption is another metabolic change characteristic of rapidly proliferating cells [75]. Interestingly, the increase glutamine demand of proliferating cancer cells does not reflect an increase of the amino acid pool for protein synthesis [76]. Instead, rapidly proliferating cancer cells use glutamine for other important tasks, e.g. to synthesize anti-oxidative glutathione, maintain cellular pools of NADPH, and fuel anaplerotic reactions to replenish tricarboxylic acid (TCA) cycle intermediates [76, 77]. Glutaminolysis is the process of glutamine conversion to glutamate by glutaminase (GLS) and, subsequently, to a-ketoglutarate (a-KG), which enters the TCA cycle to contribute to amino acid, nucleotide, and fatty-acid biosynthesis. Glutaminolysis is elevated in cancer cells. In order to meet their increase needs for glutamine, cancer cells upregulate the glutamine transporter Solute Carrier Family 1 member 5 (SLC1A5) in a Myc-dependent manner [78, 79]. In addition, oncogenic Myc increases glutamine uptake and the conversion of glutamine into a mitochondrial carbon source by promoting the expression of GLS [54]. Myc also promotes glutamine import by inducing the glutamine transporter ASCT2 [78]. The reliance on glutamine metabolism appears especially critical under metabolic stress conditions, particularly under glucose and oxygen deprivation. Interestingly, Myc overexpression has been shown to be sufficient to induce glutamine “addiction,” i.e. cancer cells dependence on glutamine metabolism for survival [80].

Besides the contribution to amino acid biosynthesis as basic building block for protein synthesis, glutamine has another role in the cancer cell, which involves protein translation. Abundant glutamine levels modulate mTORC1, a key regulator of protein translation [81]. In the presence of sufficient amounts of amino acids [Glutamine and essential amino acids (EAA)], growth factor signaling through PI3K-Akt or the extracellular signal-regulated kinase (ERK)-ribosomal protein S6 kinase (RSK) pathways activate mTORC1 [82]. A portion of the imported glutamine into cells is not utilized for anabolic metabolism but rather shuttled out of the cell in exchange for EAAs that can be used for protein synthesis. Therefore, glutamine serves as precursor for protein synthesis, as an inducer of mTORC1 signaling, and as a source of EAAs to promote protein translation [82].

#### Redox status (ROS/NADPH/GSH/TRX)

The high metabolic activity of cancer cells results in elevated ROS levels. Cancer cells develop several protective mechanisms to counteract the toxic effects of high ROS by increasing the production of metabolites that have reductive power. Amino acids can fuel macromolecule production, but at the same time particular amino acids, i.e. glutamine, are utilized for the generation of NADPH. The cellular NADPH pool is also sustained by PPP, through IDH1/2-mediated conversion of isocitrate to αKG, and by ME1-mediated conversion of malate to pyruvate [10]. NADPH is either used as a cofactor or as a reducing agent. The latter function is of particular importance since rapidly proliferating cells produce ROS as byproduct in many anabolic processes. As a crucial antioxidant, NADPH provides the reducing power for both the glutathione (GSH) and thioredoxin (TRX) systems that scavenge ROS and repair ROS-induced damage [83]. Glutamine through aKG serves the de novo production of GSH [84]. GSH reduces ROS by serving as an electron donor via its thiol group. During this process two molecules of glutathione are converted to the oxidized form, glutathione disulfide (GSSG). Once oxidized, GSSG can be reduced by glutathione reductase in a NADPH-dependent manner [84]. Similar to GSH, thioredoxins act as antioxidants by mediating cysteine thiol-disulfide exchange. Oxidized thioredoxins are converted back to the reduced state by the NADPH-dependent flavoenzyme thioredoxin reductase [85]. Another mechanism by which cancer cells counteract elevated ROS levels is based on the transcription factor nuclear factor erythroid 2-related factor 2 (NRF2) [86]. NRF2 is the master regulator of intracellular antioxidant responses. Hyperactive PI3K signaling [87] as well as mutations in the NRF2 gene [88] lead to the constitutive stabilization of NRF2 causing expression of anti-oxidant genes like glutathione reductase [89, 90], thioredoxin [91], thioredoxin reductase 1 [89, 90], and others [86]. It is also noteworthy that in tumor cells NRF2 can enhance expression of genes involved in PPP such as glucose-6-phosphate dehydrogenase, phosphogluconate dehydrogenase, transketolase, and transaldolase 1, which contribute to NADPH regeneration [92].

Peroxisome proliferator-activated receptor gamma coactivator 1-alpha (PGC-1α) is a transcriptional coactivator known as a key regulator of mitochondrial biogenesis and function [93]. Nevertheless, it was recently shown that PGC-1α is upregulated in melanoma and protects cancer cells from ROS by enhancing the expression of ROS detoxifying genes [94]. The guardian of the genome, p53, also contributes to the cellular ROS balancing [95]. However, the function of p53 in the generation and detoxification of ROS is complex and can have opposing effects. p53 may cause the generation of ROS by the induction of a gene cluster named PIG1–13 (p53-inducible genes 1–13), which has strong pro-oxidant properties [96]. It is likely that this feature of p53 is related to its pro-apoptotic function. However, ROS are particularly potent in promoting DNA damage and p53-dependent ROS detoxification is part of the cellular DNA damage response. Upon ROS-mediated damage p53 enhances the transcription of several antioxidant genes [97], including Sestrins [98], glutathione peroxidase 1 (GPx1) [99], and TIGAR [46]. Therefore, the effect of p53 on balancing cellular ROS in cancer cells probably depends not only on the presence or absence of p53 but also on the particular mutation of p53, which might alter the preference of p53 for the regulation of distinct target genes [100].

An additional mechanism for NADPH generation depends on pyruvate kinase (PK). PK catalyzes the rate-limiting, ATP-generating step of glycolysis in which phosphoenolpyruvate is converted to pyruvate [101]. In cancer cells the M2 isoform (PKM2) is specifically upregulated. However, PKM2 is typically found in an inactive state and is less efficient in promoting glycolysis [102–104]. Despite slowing glycolysis, PKM2 provides a growth advantage to cancer cells. PKM2 allows carbohydrate metabolites to enter other pathways, which generate macromolecule precursors required for cell growth and reducing equivalents such as NADPH, which maintain redox balance [10]. At the molecular level, PKM2 can be regulated by oncogenes. Specifically, the oncogene c-Myc promotes PKM2 over PKM1 by modulating exon splicing [105]. Additionally, PI3K and mTOR signaling can increase PKM2 expression through HIF1α-regulated transcription [106, 107]. In addition to glylcolysis, PKM2 is also involved in regulating gene expression and cell cycle progression. Nuclear PKM2 can interact with β-catenin to enhance its activity, implicating PKM2 as a transcriptional co‐activator [108]. Furthermore, PKM2‐dependent histone H3 phosphorylation can induce gene expression, cell proliferation and tumorigenesis [109, 110].

#### Epigenetics

Epigenetic alterations are well-known causes for the onset of cancer. These include global genomic DNA hypomethylation, site-specific CpG island changes, as well as altered histone modifications and non-coding RNAs. Most of these changes can be caused by mutations in the enzymes involved in these processes. Nevertheless, epigenetic modifiers rely on cofactors to generate epigenetic marks. These cofactors are generated via different cellular metabolic pathways. Therefore, one can imagine that global deregulation of cellular metabolism can influence epigenetic enzymes by the amount of cofactors present in a cell. Also, accumulation of inhibitory or activating molecules can change the enzymatic activity of epigenetic modifiers and consequently the cellular epigenome. For example, succinate, fumarate, and 2-hydroxyglutarate can inhibit the activity of histone and DNA demethylases (HDMs and DDMs) at high concentrations and can act as oncometabolites, i.e. metabolites that can promote tumorigenesis by altering the epigenome [111]. 2-Hydroxyglutarate (2HG) is generated by mutated IDH and is commonly found in gliomas and acute myeloid leukaemia but not in the normal counterparts of these cancer cells [112]. These oncometabolites are structurally similar to their precursor metabolite, α-ketoglutarate. As a consequence, they act as competitive inhibitors for a superfamily of enzymes called the α-ketoglutarate-dependent dioxygenases. These enzymes function in fatty acid metabolism, oxygen sensing, collagen biosynthesis, and modulation of the epigenome [113]. Several tumor types with succinate dehydrogenase (SDH) mutations have elevated levels of succinate and fumarate and have been identified to carry characteristic DNA hypermethylation patterns, which are due to reduced DDMs activity. Particularly, the DDM 10-11-translocation methylcytosine dioxygenase (TET), which removes methyl groups in an αKG-dependent manner, is inhibited. Such alterations have been shown to be sufficient to drive oncogenesis [114, 115]. The jumunji-C HDMs are a major class of α-KG-dependent dioxygenases [116]. They initiate the removal of methyl groups from histones by hydroxylation and thus alter gene transcription. In tumors with IDH1/2 mutations, high levels of fumarate, succinate, and 2HG have been reported, and these increases correlate with elevated histone methylation for H3K9 and H3K27, which are gene repressive marks [117].

Recent studies have shown that metabolic flux through the TCA cycle can affect gene transcription and/or epigenetic programs. For example, TCA intermediates such as α-KG, succinate, and fumarate can directly or indirectly affect the activities of metabolic enzymes, transcription factors such as HIF1α, and epigenetic regulators such as histone demethylases [109, 118–120]. Interestingly, α-KG has also been involved in the maintenance of pluripotency of embryonic stem (ES) cells by epigenetic mechanisms [121]. Moreover, in ES cells glucose availability and acetyl-CoA production can also influence epigenetics by regulating the cytosolic acetyl-CoA pool available for histone acetylation reactions [122, 123], raising the possibility that such mechanisms might be operable in cancer and particularly in cancer initiating cells, which have stem cell-like properties. Epigenetic regulation of gene expression and function via altering histone/DNA methylation and acetylation levels and their therapeutic targeting is currently a rapidly developing field and will likely provide new opportunities to target the consequences of metabolic reprograming in tumors and potentially immune cells.

#### Changes in the microenvironment

All the above-mentioned intracellular metabolic processes, which are altered in cancer cells, affect the tumor microenvironment by several means. The consequences of such changes have significant impact on cancer cells as well as non-cancerous cells present in the tumor microenvironment.

#### Hypoxia

Hypoxia arises in tumors through the uncontrolled proliferation of cancer cells. Due to the rapid proliferation and lack of sufficient vascularization, cancer cells quickly exhaust the oxygen (and nutrient) supply from the normal vasculature and create a hypoxic microenvironment [124]. Under these conditions the adaptation to glycolysis described above provides a strong selective growth advantage of tumor cells over non-tumor cells. Under hypoxic conditions the transcription factor HIF1α is stabilized [125]. Moreover, the oncogene-activated PI3K pathway can also stabilize HIF1α, even under normoxic conditions [126, 127]. Upon activation, HIF1α triggers transcription of glucose transporters and glycolytic genes [128]. HIF1α can also decrease the rate of OXPHOS by reducing the flux of pyruvate into the TCA cycle through fostering transcription of pyruvate dehydrogenase kinases [129, 130]. It is of note that under physiological conditions HIF1 can inhibit the activity of Myc. Nevertheless, oncogenic Myc collaborates with HIF to augment aerobic glycolysis [131]. It has been found that high levels of Myc bind to a new set of target genes, suggesting that the behavior of Myc depends on its level [132, 133].

#### Nutrient deprivation

In addition to the deprivation of oxygen, the fast growing cancer cells also consume most nutrients from the surrounding. High demand for energy and anabolic metabolism, increased import, and fast metabolic flux cause nutrient deprivation to other cells residing in the same niche. Besides its direct effect on metabolism, nutrient deprivation causes endoplasmic reticulum (ER) stress in the microenvironment. In order to counteract the ER stress-associated damages, several cellular processes, collectively named “unfolded protein response” (UPR), are activated. The UPR has a dual function: it attenuates ER-associated damage, and if this is not feasible, it activates apoptosis [134]. Additionally, nutrient deprivation can cause autophagy, which is a highly regulated process involving lysosomal degradation of intracellular components, damaged organelles, misfolded proteins, and toxic aggregates. Autophagy can be induced in response to various conditions, including nutrient deprivation, metabolic stress, and hypoxia to adapt cellular conditions for survival. However, extensive autophagy-based degradation pathways may cause autophagy-associated cell death [135]. Thus, cells in the microenvironment are challenged by the cell-death-inducing effects of nutrient deprivation.

#### Metabolic waste

**Fig. 2:**
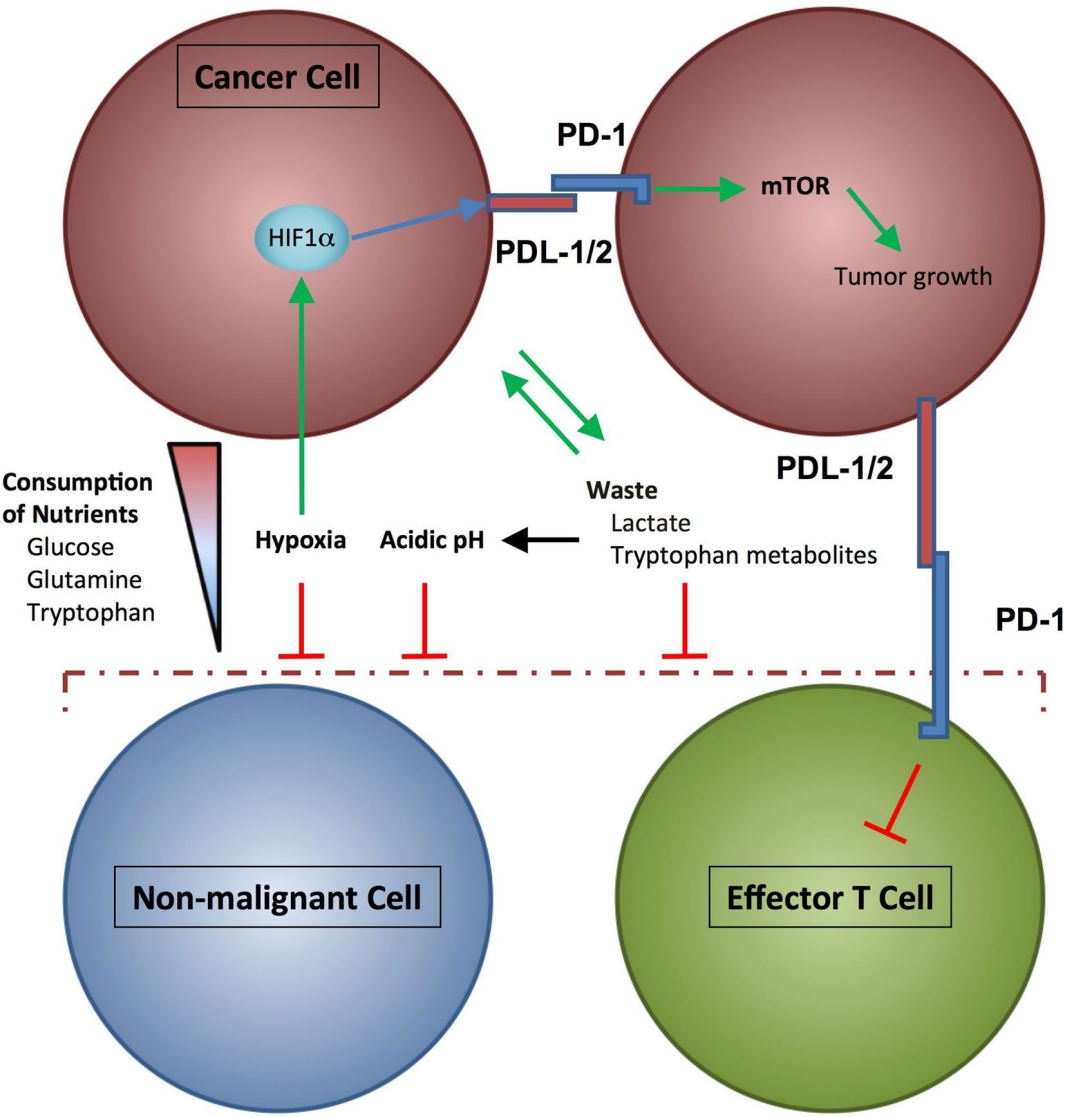
Cancer cells induce several metabolic changes in the microenvironment. The increased uptake of nutrients such as glucose and amino acids depletes these resources for non-tumor cells. This can inhibit the growth and function of non-tumor cells in the microenvironment due to the lack of resources for cellular metabolism. Moreover, nutrient depletion can lead to autophagy, ER-stress, and, finally, to cell death. Cancer cells also generate a hypoxic microenvironment. The prolonged lack of oxygen inhibits regular cell function in non-tumor cells, whereas in cancer cells hypoxia (in combination with oncogenic PI3K signaling) stabilizes HIF1α and promotes the glycolytic phenotype. Additionally, HIF1α enhances expression of PDL-1, which can engage with PD-1 on other cancer cells in the microenvironment. This activates mTOR signaling and supports tumor growth, whereas engagement of PD-1 in T cells inhibits T cell activation and growth. Thereby, cancer cells can spread an inter-cancer-cell growth signal, while suppressing responses of tumor-infiltrating T cells. The increased metabolic activity of cancer cells produces waste byproducts, like lactate and tryptophan metabolites, which are secreted to the microenvironment and act inhibitory on non-tumor cells. Lactate not only acidifies the microenvironment resulting in inhibition of the surrounding non-tumor cells, but can also be re-imported into cancer cells and can be used to feed into glycolysis

As a consequence of their high metabolic rate, tumor cells generate high amounts of metabolic “waste,” which is transported out of the cell. Accumulation of these products creates a harsh and potentially metabolically toxic environment. For example, increasing amounts of lactate acidify the microenvironment. Interestingly, cancer cells can consume lactate as a metabolic fuel and utilize it through conversion back to pyruvate and subsequent oxidation to provide a fuel in times of nutrient depletion [136]. Various downstream metabolites generated following tryptophan breakdown are also released by cancer cells into the microenvironment. Cells in the tumor microenvironment take up kynurenine (Kyn) via the amino acid transporter LAT1. Subsequently, Kyn binds to the aryl hydrocarbon receptor (AhR), which translocates from the cytoplasm to the nucleus, where it can drive expression of various genes [73]. Kynurenic acid can enter cells via the human organic anion transporters hOAT1 and hOAT3 [137–139]. Quinolinic acid is an *N*-methyl-d-aspartate receptor agonist, whereas kynurenic acid is an antagonist of this receptor [140]. These findings suggest that tryptophan-derived metabolites can affect functionality of cells in the tumor microenvironment. The hypoxic, acidified, and nutrient-deprived environment causes metabolic stress to neighboring non-malignant cells and acts as a barrier shielding the tumor from any impact mediated by tumor-specific T effector cells residing or recruited in the tumor microenvironment (Fig. 2).

#### Basic metabolic features of T cells

**Fig. 3:**
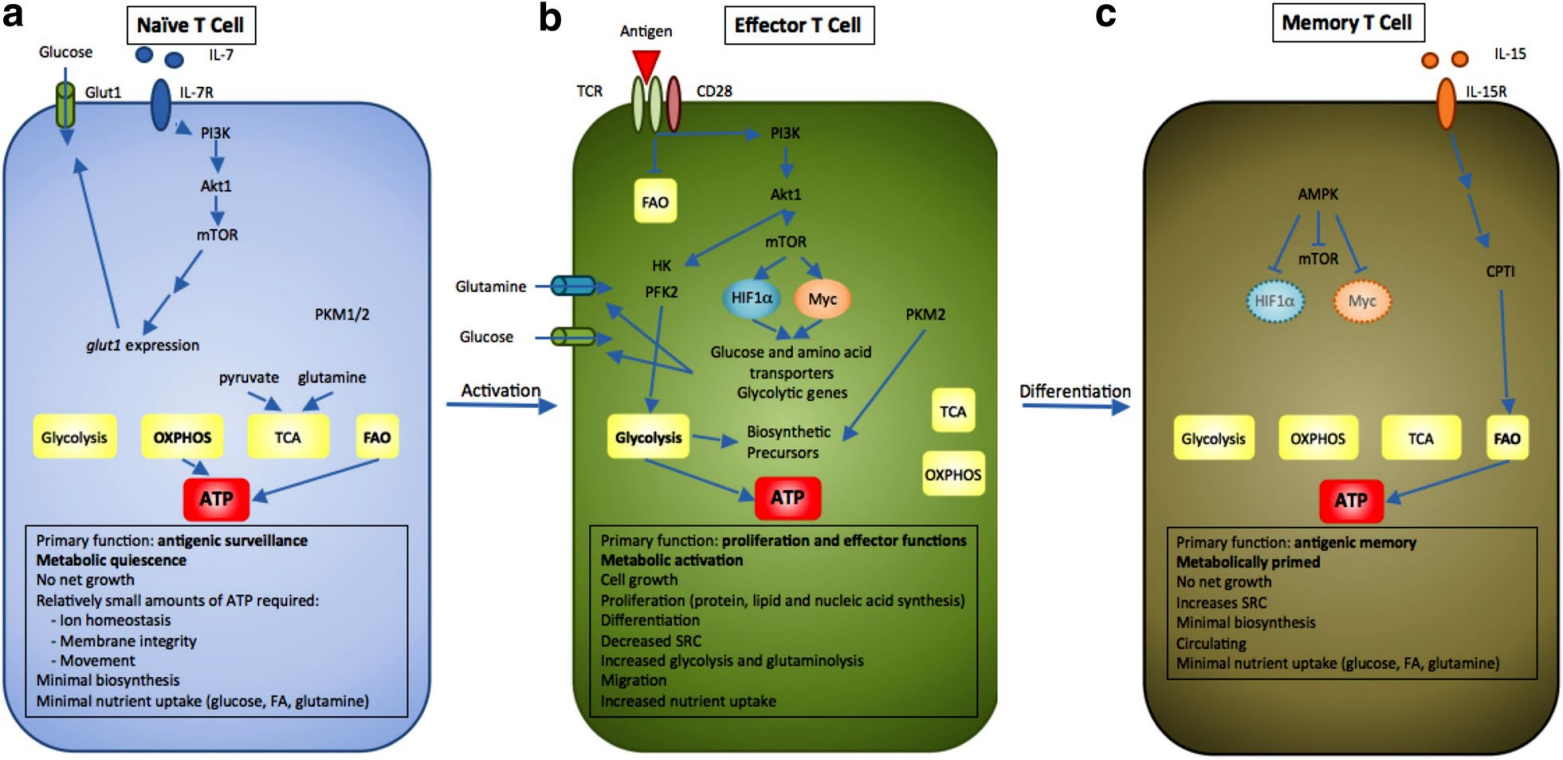
T cell differentiation is accompanied by metabolic changes, which can be affected by the tumor microenvironment altering their fate and function. **a** Naïve T cells function in antigenic surveillance and do not proliferate. This requires minimal energetic and biosynthetic activity which is represented by a metabolically quiescent state which is accompanied by minimal nutrient uptake. The only energy-demanding processes are ion homeostasis, membrane integrity, and movement. The primary ATP sources are OXPHOS and FAO to fuel the low energy demand. IL-7 signaling and activation of PI3K-Akt1-mTOR is required for survival and basal, low level Glut1 expression. (Furthermore, naïve T cells express low levels of both isoforms of PKM1 and PKM2 keeping PKM2 oncogenic function in check. Low quantities of pyruvate and glutamine are utilized in the TCA cycle. **b** Upon antigen encounter T cells differentiate into effector cells. This process is accompanied by metabolic changes which are required to fulfill the new effector functions and rapid proliferation. Antigen binding to the TCR and co-activation by CD28 inhibits FAO and activates PI3K-Akt1. This activation triggers glycolytic enzymes HK and PFK2. Additionally, mTOR signaling is turned on which enhances expression of glycolytic genes, glucose, and amino acid transporters via activation of transcription factors HIF1α and Myc. Effector T cells also switch from balanced PKM1 and PKM2 expression to increased and predominant expression of PKM2, which promotes generation of biosynthetic precursors. Additionally, the SRC is decreased and the uptake of nutrients is enhanced. These events promote the establishment of a glycolytic phenotype with increased glutaminolysis combined with a high degree of protein, lipid, and nucleic acid synthesis to support cell growth and proliferation. **c** After antigen challenge most effector T cells undergo apoptosis during the contraction phase. A small proportion differentiates into memory T cells with prolonged survival capacity to provide long-term antigenic memory. Memory T cells do not proliferate and thus have minimal biosynthesis and nutrient uptake. However, they show increased SRC, which supports their ability to rapidly proliferate upon re-encounter of antigen. This cellular fate includes another metabolic adaption. In particular, metabolic switch to FAO via increased CPT1 expression and elevated AMPK activity, which represses HIF1α, mTOR, and Myc. Thereby, AMPK inhibits glycolysis, which was the primary ATP source during the effector phase. Extracellular queues that support memory cell formation -such as IL-15- promote these metabolic changes. Naïve T cells can differentiate into different subsets of specialized T cells mainly depending on extracellular stimuli and factors. The tumor microenvironment influences these cell fate decisions in a metabolic manner. The lack of glucose, amino acids, and oxygen as well as the accumulation of metabolic byproducts secreted to the microenvironment generate a milieu that suppresses glycolysis-dependent T cell fates like CD8^+^ T effector cells and CD4^+^ Th1/2/17. In contrast, FAO-related cell fates such as CD8^+^ T cell memory and CD4^+^ T regulatory cells are promoted. Especially, amino acid depletion supports formation of immunosuppressive macrophages. Taken together, the tumor microenvironment generates an immunosuppressive milieu that fosters immune evasion

Naïve T cells have low metabolic requirements and depend predominantly on the utilization of low amounts of various nutrients including glucose, fatty acids, and amino acids, as well as on pyruvate and glutamine oxidation via the TCA cycle (Fig. 3). In order to maintain this basal energy-generating metabolism to support their continuous migration through secondary lymphoid tissues and immune surveillance, naïve T cells require cell extrinsic signals such as IL-7 [9, 141]. Upon antigen encounter there is a dynamic change on T cell metabolism characterized by extensive proliferation and differentiation into effector T cells (T_EFF_). TCR-mediated signaling promotes the upregulation of glucose and amino acid transporters at the T cell surface and directs the metabolic reprogramming of naïve T cells from OXPHOS to glycolysis, which is mandatory for the acquisition of T cell effector differentiation and function [9, 142]. During the subsequent stages of T cell response, when the pathogen is cleared, most Teff cells undergo cell death resulting in a dramatic reduction of antigen-specific Teff -a process known as contraction phase- leaving behind a small population of antigen-specific T cells, which survive this demise and become T memory cells (Tm). Tm cells display a characteristic increase in mitochondrial mass and thus a greater mitochondrial spare respiratory capacity (SRC) [9, 143, 144], which is the maximal mitochondrial respiratory capacity available to a cell to produce energy under conditions of increased work or stress.

For energy generation, Tm cells rely on β-oxidation of de novo generated fatty acids that have been synthesized from glucose during the effector phase by fatty acid synthesis and have been stored intracellularly, instead of uptaking and using extracellular lipids [145]. Interestingly, for membrane biosynthesis, lymphocytes also rely on de novo generated free fatty acids from glucose and glutamine, despite the availability of extracellular lipids [145–147]. In contrast to the dominant role of fatty acid oxidation (FAO) in memory cells for energy generation, FAO and other ATP-generating catabolic pathways are actively suppressed in Teff cells. A key mechanism responsible for this outcome is mediated by the transcription factor Myc, which is induced during T cell activation and has a dominant role in driving metabolic reprogramming by promoting glycolysis and glutaminolysis and suppressing FAO and other ATP-generating catabolic pathway [148]. Importantly, Myc also has a role in the fate of T cells after division, which is induced during encounter of T cell with APC [149]. Specifically, upon activation by antigen-presenting cells (APCs), T cells can undergo asymmetric cell division, wherein the daughter cell proximal to the APC is more likely to differentiate into an effector-like T cell and the distal daughter is more likely to differentiate into a memory-like T cell [150]. Myc has an active role in regulating the asymmetric distribution of amino acid transporters, amino acid content and mTORC1, which also correlated with an asymmetric glycolytic metabolic phenotype between the two daughter cells. Thus, Myc regulates metabolic programs not only at the early stages of T cell activation but also after daughter cell division.

Under sufficient energy conditions, intermediate metabolites of glycolysis can be used in the PPP to support nucleotide biosynthesis and NADPH production important for anabolic pathways and redox control. Moreover, glycolytic enzymes have been linked directly to the regulation of T cell function. For example, when not engaged in glycolysis, the glycolytic enzyme glyceraldehyde 3-phosphate dehydrogenase (GAPDH) binds to AU-rich elements within the 3′ UTR of IFN-γ mRNA and prevents translation of IFN-γ mRNA, thereby suppressing IFN-γ production [19]. Another example involves the glycolytic enzyme pyruvate kinase (PK), which catalyzes the conversion of phosphoenolpyruvate (PEP) to pyruvate during glycolysis. Differential splicing generates two isoforms, M1 or M2. With the PKM2 splice variant being expressed in embryonic tissues, proliferating cells, and tumor cells PKM2 coordinates glycolytic flux and cell proliferation [61, 104]. Because the M2 isoform is less efficient in converting PEP to pyruvate than the M1 isoform, it has been suggested that this property slows down glycolysis towards lactate but skews glycolysis towards biosynthetic pathways, thus giving cells a growth advantage [151]. Resting naïve T cells express both M1 and M2 isoforms, while mitogen activation promotes the rapid accumulation of the M2 isoform, which becomes the dominant isoform expressed in Teff cells [152, 153]. In addition, despite its relatively low affinity for PEP, dimeric PKM2 is able to use PEP as a phosphate donor and catalyzes the in vitro phosphorylation of some protein targets, including the transcription factor STAT3.

## Role of metabolism in T cell differentiation

Many recent studies have shown that distinct T cell fates can be imprinted in divergent metabolic programs. For example, the metabolism-cell fate connection has been shown with the switch to glycolysis that accompanies Teff differentiation and the switch to FAO that accompanies the conversion of Teff to Tm [154]. Also, enforcing FAO by elevating AMPK activity or by inhibiting mTOR results in increased numbers of Tm cells [154, 155], which are capable of regenerating the specific T cell clone upon encounter with the same antigen. This ability of memory T cells for clone regeneration is reminiscent of the properties of the very small fraction of OXPHOS-dependent cancer cells with stemness features, which resides within the tumors, as mentioned above. In addition, conversion toward a T regulatory (Treg) phenotype is also favored in conditions of increased OXPHOS and decreased glycolysis. Although pronounced differences have been established between the metabolic programs of Teff and Treg cells, distinct metabolic differences within the various Teff subsets have not yet been discovered. At the metabolic level, Teff cells Th1, Th2, and Th17 cells and Treg are the best-defined CD4^+^ T cell subsets. It is well known that a variety of cytokines can determine the differentiation fate of T cell subsets [156–160], and it is becoming increasingly clear that metabolism plays a significant role in driving these distinct differentiation programs. For example, there is a strong bias toward glycolysis over mitochondrial metabolism by proinflammatory CD4^+^ Th1, Th2, and Th17 lineages whereas induced CD4^+^ Treg lineage cells display a mixed metabolism involving glycolysis, lipid oxidation, and OXPHOS [18]. It is noteworthy that blockade of glycolysis during in vitro Th17 differentiation favors Treg formation rather than Th17 cells [161]. Addition of exogenous fatty acids (FAs) in the culture of T cells activated under skewing conditions strongly inhibits the production of Th1, Th2, and Th17 cytokines, but not the Treg suppressive function. Importantly, inhibition of Teff function in the presence of FAs cannot be rescued by re-addition of Th1-, Th2-, and Th17-promoting cytokines [18].

## Effects of the tumor microenvironment on T cell fitness

### Exhaustion

T cell “metabolic fitness” is central to effective antitumor immunity but is compromised by unique conditions of limited nutrient availability in the tumor nutrient microenvironment and the effects of immune checkpoints [162]. Within the immune suppressive tumor microenvironment, T cells acquire an “exhausted” phenotype, which is characterized by progressive loss of effector functions, changes in expression and function of key transcription factors, and metabolic alterations. In addition “exhausted” T cells are characterized by a sustained upregulation and co-expression of multiple inhibitory receptors [163, 164]. The multiple metabolic constraints imposed by the cancer cell metabolism in the tumor microenvironment minimize the potential of T cells to mediate effector function. It is likely that nutrient availability, effects of inhibitory receptors and metabolic changes of the microenvironment drive T cell differentiation to the state of exhaustion. T cells receiving signals through the key inhibitory receptor PD-1 are switched to low glycolytic and low OXPHOS state with limited biosynthetic activity and low antioxidant reserves [15]. It is likely that such changes in metabolic reprogramming drive T cell differentiation to the state of exhaustion. T cell exhaustion is an active process and can lead to measurable consequences on T cell function. For example, distinct subsets of exhausted T cells exist with different potentials for recovering function after blockade of the PD-1 pathway. Exhausted T cells with intermediate expression of PD-1 (PD-1^int^) can be reinvigorated by blockade of the PD-1 pathway, whereas those with high expression of PD-1 (PD-1^high^ cells) cannot [163, 164]. These distinct subsets of exhausted T cells may also have different bioenergetic properties and differential capacity for mitochondrial biogenesis. It is tempting to speculate that the degree of exhaustion and the ability to reinvigorate exhausted T cells might depend on the reserve of lipids, which appear to be the only source of energy generation by FAO in T cells receiving PD-1 signals [15]. It is also possible that blockade of the PD-1: PD-L1 pathway may lead to transient regeneration of Teff cells if the metabolic defects of exhausted tumor-specific T cells are not fully recovered. Thus, identification of the distinct bioenergetic profiles in exhausted T cell subsets might provide new tools to determine the level of T cell exhaustion and also identify novel targets to reverse exhaustion in addition to PD-1 blockade.

### Nutrients

The main nutrients that support survival and growth of T lymphocytes are glucose, amino acids, and lipids, and their deprivation results in impaired T cell function. Depending on the type of nutrients present, T cells can acquire distinct differentiation program and functional fate [21, 165, 166]. In the tumor microenvironment cancer and T cells develop metabolic competition for the utilization of available nutrients among which glucose has a central role. Cancer cells are addicted to a steady state glycolysis, whereas T cells depend on glucose during the process of antigen-mediated activation that leads to expansion and development of effector functions. Limiting glucose in the culture decreases the activation of naïve T cells and the production of effector cytokines although a significant degree of proliferation might be preserved through OXPHOS [19, 148, 167–172]. Mechanistically, glycolysis might promote IFN-γ production by inducing GAPDH activation and releasing GAPDH from binding at the 3′UTR of the IFN-γ promoter, thereby inducing IFN-γ production via post- transciptional mechanisms [19]. In addition, the glycolytic metabolite phosphoenolpyruvate (PEP) promotes T cell activation by sustaining T cell receptor-mediated Ca^2+^-NFAT signaling and effector functions due to repressing sarco/ER Ca^2+^-ATPase (SERCA) activity [17]. Because glycolysis is intimately linked with T effector differentiation, glucose deprivation due to high glucose flux in the cancer cells results in limited glucose availability for utilization by T cells. As a consequence, T cells not only are unable to develop tumoricidal effector functions but also alter their differentiation program resulting in the generation of cell types that develop during limited glucose supplies, such as Treg and T exhausted T cells [15, 18].

T cells also activate glutamine transport and catabolism mechanisms during their stimulation [167–169]. Glutamine has an essential role to support the TCA cycle through anaplerosis after prior conversion to glutamate and aKG and is also critical for ATP production via OXPHOS even in presence of glucose [20]. Glutamate also serves as a key component for glutathione (GSH) synthesis and antioxidant defense [173]. Similarly to glucose, cancer cells use glutamine for their metabolic needs [174] indicating that competition for glutamine utilization by cancer and T cells also occurs in the tumor microenvironment. Therapeutic targeting of glutamine metabolism by glutaminase inhibitors in cancer cells is currently under investigation in clinical trials for hematologic malignancies and solid tumors (https://www.clinicaltrials.gov/). It remains to be determined how such therapeutic approaches will affect T cell differentiation and function.

Decreased levels of the essential amino acid, l-tryptophan, can be caused by overexpression of IDO on tumor cells [175, 176]. It is well documented from studies in mouse models and patients that IDO expressed in cancer cells or DC in the cancer microenvironment mediates immunosuppressive effects and tolerance to tumor via mechanisms independent from l-tryptophan deprivation [177, 178]. Detailed recent studies designed to delineate the mechanisms of tumor cell IDO-induced immunosuppression showed that the effect of IDO in the tumor microenvironment is mediated through increased differentiation of Treg cells at the tumor site, systemic expansion of myeloid cells and marked recruitment of myeloid-derived suppressor cells (MDSCs) into the tumor microenvironment [179]. Such correlation between IDO expression and MDSC infiltration has been identified not only in experimental animal models but also in samples from human patients with melanoma [179]. Metabolic competition and nutrient deprivation, accumulation of metabolic byproducts, effects of IDO act in concert with microenvironmental changes induced by hypoxia and tumor aerobic glycolysis to form a barrier to antitumor T cell immunity.

### Lactate

Lactic acid is largely produced by highly glycolytic cancer cells and can suppress the proliferation and cytokine production of human cytotoxic T lymphocytes (CTLs) and reduce cytotoxic activity [180, 181]. Activated T cells also secrete lactate but, due to their higher metabolic rate, cancer cells are most likely the key source of lactate in the tumor microenvironment. Moreover, since PD-1 ligation results in suppression of glycolysis and diminished lactate production [15], engagement of PD-1 in T cells, in the context of cancer, will mitigate glycolysis and production of lactate. Thus, the main source of lactate is the tumor, and eliminating lactate production by tumor cells would be anticipated to improve T cell mediated antitumor immunity. A major consequence of lactate secretion is microenvironmental acidification. Studies that have addressed the role of acidic pH on immune function in the context of tumor have mostly focused on the impact of lactate-mediated acidification on macrophage polarization to the M2 suppressive phenotype [182]. However, lactate-mediated acidification can also induce Arginase 1, which promotes the depletion of extracellular arginine levels, resulting in inhibition of efficient T cell proliferation and activation [182, 183]. Moreover, manipulation of the pH of the tumor microenvironment by the use of proton pump inhibitors results in less dysfunctional tumor infiltrating lymphocytes and increased therapeutic efficacy of both active and adoptive immunotherapy [184]. Despite its negative impacts on the immune cells, certain cancer cell types can utilize lactate itself as a metabolic fuel through reverse conversion to pyruvate that is subsequently oxidized to provide a fuel in times of nutrient depletion [136]. The extent to which T cells can oxidize lactate is unknown. It is also unknown whether such biochemical pathway might impact selectively on Teff, Tm, or Treg differentiation and/or function.

### Hypoxia

Hypoxia occurs physiologically in the microenvironment of primary lymphoid organs, bone marrow, and thymus [185, 186] and plays a critical role in immune cell function and development [187, 188]. However, most prevalently, hypoxia is the hallmark of tissue microenvironments under pathological conditions as in the cases of cancer, inflammation, infection, necrosis, and autoimmunity. Under the survival- and growth-unfavorable conditions of the hypoxic microenvironment, all cells, including T lymphocytes, need to adapt metabolic programs that support their survival [148, 189, 190]. Hypoxia inducible factors (HIFs) are the main transcription factors that sense and respond to hypoxia with HIF-1α and HIF-2α being the most widely studied members [191]. The activity of HIFs is regulated by post-transcriptional modifications, which involve the hydroxylation of their proline and asparagine residues prolyl hydroxylases [192]. The von Hippel–Lindau tumor suppressor (VHL) protein binds to hydroxylated HIF1α targeting it for ubiquitination and degradation by the proteasome. In normoxic conditions, hydroxylation and proteasomal degradation of HIF1α subunits take place, whereas under hypoxic conditions the hydroxylases are no longer active, allowing HIF1α to translocate to the nucleus and bind to HIF1β in order to regulate the transcription [193].

The hypoxia response, which is based on the function of the HIF1α and HIF2α transcription factors, upregulates glucose transporters and multiple enzymes of the glycolytic pathway [128, 194]. In contrast, HIFs negatively regulate TCA and OXPHOS regulatory genes. In renal carcinoma cells lacking VHL protein, the main negative regulator of HIFs, HIF1 negatively regulates mitochondrial biogenesis and oxygen consumption by inhibiting the activity of c-Myc [195]. Similarly to its effect in tumor cells, HIF1α mediates the metabolic switch from OXPHOS to aerobic glycolysis in lymphocytes [161] and directly regulates the Th17/Treg balance in favor of Th17 cells [196]. HIF1-deficient T cells display a marked reduction in the expression of IL-17 and Th17-signature genes. Moreover, mice with HIF1α-deficient T cells are resistant to induction of Th17-dependent experimental autoimmune encephalitis, and this outcome is associated with diminished Th17 and increased Treg differentiation [196]. HIF1α activates Th17 development through RORγt and p300, and also attenuates Treg development by targeting Foxp3 for proteasomal degradation [161]. As a consequence, blocking glycolysis during Th17 cell differentiation reduces the development of Th17 cells and favors Treg formation [161], suggesting a direct link between metabolism and T cell fate determination. Conversely, deletion of VHL, which leads to elevated levels of HIFs, results in differentiation of cytotoxic CD8^+^ lymphocytes (CTLs) with a high glycolytic and low OXPHOS activity and, thus, favors the differentiation of effector CD8^+^ T cells [197]. It is a subject of active investigation how HIF1α affects memory formation and anti-tumor immunity. Importantly, loss of HIF1β alters expression of chemokines and receptors involved in migration and extravasation. Loss of HIF1β results in sustained CD62L expression and increased of T cell homing to secondary lymphoid organs. In addition, loss of HIF1β results in upregulation of CXCR3, CCR5, and CCR7 in CD8^+^ T cells [198]. Moreover, hypoxia may protect tumor cells from antitumor immunity and can promote HIF1α-dependent transcriptional upregulation of PD-L1 on cancer cells and myeloid-derived suppressor cells (MDSCs) that may inhibit PD-1-expressing T cells [199, 200]. Indeed, tumor cells have greater resistance to T cell-mediated killing under hypoxic conditions [200]. Thus, hypoxia affects both tumor and T cells and shapes the expression of critical transcription factors, effector molecules, chemokines, chemokine receptors, costimulatory receptors, and activation-induced inhibitory receptors in a HIF-dependent manner. Therapeutic strategies targeting HIFs in the immune system might be beneficial for anti-tumor immunity.

### Reactive oxygen species

Besides being considered as harmful by-products of metabolism or weapons of phagocytes against pathogens, ROS can also serve as signaling messengers in a multitude of pathways. It has also become evident that the source, kinetics and localization of ROS production all influence cell responses [201–204]. Tumors are influenced by changes in ROS production, and previous reports actually indicate that cancer cells exhibit more oxidative stress than their healthy counterparts [205]. In particular, mitochondria-derived ROS are essential for Kras-mediated cancer cell growth [5]. In contrast, other studies showed that inhibition or abrogation of LDH-A causes a shift to higher OCR and ROS production from the mitochondria that render the tumor cells more susceptible to apoptosis [206, 207]. Moreover, it has been shown that increase in intracellular concentrations of ROS causes inhibition of the glycolytic enzyme PKM2 leading to glucose diversion into the PPP, thereby generating sufficient reducing power for detoxification of ROS [208]. In a recent article by James Watson, co-discoverer of the double helix structure of DNA, it was pointed out that causing oxidative stress by targeting highly expressed antioxidant enzymes, which are not present in non-malignant cells, holds great promise as a strategy for finding novel anti-tumor drugs. In contrast, antioxidants seem to promote tumor survival and growth [209]. Interestingly, cultured colorectal cancer cells (CRC) harboring KRAS or BRAF mutations, which upregulate the glucose transporter Glut1, are selectively killed when exposed to high levels of vitamin C, but this is not due to the antioxidant function of vitamin C. Instead, this effect is mediated by the increased uptake of the oxidized form of vitamin C, dehydroascorbate (DHA) via the Glut1 glucose transporter, which is subsequently reduced to vitamin C, thereby depleting glutathione and causing oxidative stress. Thus, in cancer cells ROS accumulates and inactivates GAPDH leading to an energetic crisis and cell death not seen in CRC cells not harboring KRAS and BRAF mutations [210].

In T cells, ROS can be derived from the catalytic activity of NADPH oxidase 2 (NOX-2), which is a catalytic subunit of phagocyte oxidase (PHOX) expressed in the plasma membrane of T cells, or from dual oxidase I (DUOX-1), which is a cytoplasmic non-phagocytic isoform of NADPH oxidase [204, 211]. ROS can be generated from the electron transport chain of mitochondria [212, 213] as well as by lipoxygenases [214]. T cell activation is paralleled by transient generation of low, physiologically relevant levels of ROS, which facilitates activation of ROS-dependent transcription factors, NF-kB and AP-1 [215, 216]. This oxidative signal is indispensable for T cell activation. Together with a Ca^2+^ influx, it constitutes the minimal requirement for activation-induced gene expression (e.g., interleukin 2 [IL-2], IL-4, CD95 ligand) [203]. In contrast to the indispensable role of low ROS levels in T cell activation, prolonged exposure to high ROS concentrations can inhibit T cell proliferation and lead to apoptosis [217]. In addition, incubation of T cells with reactive nitrogen species (RNS) such as peroxynitrite can inhibit proliferation [218]. Oxidative stress-induced modification to selective molecules involved in T cell receptor (TCR) signaling can render T cells hyporesponsive to activating stimuli [219]. The redox environment also affects T cell differentiation. Peripheral blood mononuclear cells (PBMC), stimulated with a ROS generator promoted Th2 and inhibited Th1 differentiation [220]. Moreover, products of lipid peroxidation, such as 4-hydroxy-2-nonenal (4HNE) and malonyldialdehyde (MDA), promote differentiation towards a Th2 phenotype [221]. Interestingly, NOX-2 deficiency leads to differentiation towards the Th17 lineage [222]. Since ROS can affect critical metabolism-related T cell signaling pathways such as the MAPKs and Akt pathways [204], it is not unexpected that ROS would directly affect T cell differentiation and function. A study of the novel mechanism of Treg-mediated suppression by extracellular redox remodeling showed that murine Tregs suppress GSH synthesis and cysteine release by DCs in a CTLA-4-dependent manner, leading to the oxidation of surface thiols, decrease in the major cellular antioxidant GSH, and reduced proliferation of conventional T cells [223, 224]. Vitamin C, a naturally occurring antioxidant, can facilitate demethylation of Foxp3 enhancer and thus promote Treg generation [225].

In a recent work from our group, metabolite analysis of T cells in the presence of the checkpoint inhibitor PD-1 showed that PD-1 ligation resulted in significantly more pronounced decrease in the levels of reduced GSH. However, T cells receiving PD-1 signals displayed higher levels of cysteine-GSH disulfide and ophthtalmate, a GSH-like product synthesized by the same enzymes. These changes indicate a higher attempt to increase GSH synthesis, which, together with the more pronounced decrease in the levels of reduced GSH, are suggestive of a more oxidative environment in T cells receiving PD-1 signals [15]. Consistent with a role of PD-1 in generating a more oxidative environment, another study showed that, following allogeneic bone marrow transplantation, alloreactive T cells simultaneously upregulated PD-1 expression and ROS production derived by FAO, resulting in higher susceptibility to metabolic inhibition by F1F0-ATP synthase complex inhibitors, and this event could be reversed by antioxidants. In that context, PD-1-blockade decreased mitochondrial H_2_O_2_ and total cellular ROS levels as well as the efficacy of ROS-dependent inhibitory modulation of F1F0-ATP synthase complex [226]. Although high ROS is toxic for T cells as it induces oxidative metabolic damage to various biochemical substrates, it should be noted that moderate levels of ROS have an indispensable role on T cell activation. The important involvement of ROS to T cell metabolic fate and function was highlighted by the recent identification of lymphocyte expansion molecule (LEM). LEM has no effects on glycolysis but controls the levels of OXPHOS complexes and respiration, resulting in the production of pro-proliferative mitochondrial ROS, which is critical for promoting antigen-dependent CD8^+^ T cell proliferation, effector function, and long-term protective memory cells in response to infection with lymphocytic choriomeningitis virus [227].

Based on the above, it is not evident whether the use of antioxidants or oxidants would be a beneficial therapeutic approach in the context of cancer. Caution should be exercised since such interventions will, most likely, also modulate T cell responses in addition to targeting cancer cells. One potential approach would be to target antioxidant genes in cancer in order to suppress the antioxidant defense mechanisms and make them susceptible to ROS-mediated apoptosis. Considering the fact that ROS are beneficial for T cell activation, a fine balance should be achieved in order to maximize anti-tumor effects without compromising T cell function.

#### Checkpoint inhibitors

During the past 5 years, cancer immunotherapy based on therapeutic targeting of checkpoint pathways has become a field of broad interest. This approach is based on the properties of T cells, which require at least two signals for activation. The first signal is mediated through TCR by recognition of specific antigen presented by the major histocompatibility complex (MHC) on antigen presenting cells (APCs). The second signal is mediated through ligation of co-stimulatory and co-inhibitory receptors, which are engaged by specific ligands expressed on APCs. The key co-stimulatory and co-inhibitory receptor-ligand pairs belong to the B7 and tumor necrosis factor (TNF) families [228, 229] although accessory molecules belonging to different families might also be important. The discovery that cytotoxic T-lymphocyte-associated protein 4 (CTLA4)- and programmed cell death protein 1 (PD-1)-dependent mechanisms provide the basis for the physiologic peripheral immunological tolerance supported the rationale that blocking the inhibitory signals mediated by CTLA-4 or PD-1 would promote activation of T cells that can recognize tumor antigens and induce anti-tumor responses. Importantly, the ligands of CTLA-4 B7-1 (CD80) and B7-2 (CD86) are expressed on antigen presenting cells (APC) but not on cancer cells, whereas the ligands of PD-1, PD-L1 and PD-L2, are expressed on APC and on cancer cells [12, 13]. The properties of these inhibitory immune checkpoint pathways leading to cancer immune evasion fostered the establishment of novel cancer immunotherapies.

Currently, there is only limited knowledge regarding the metabolic consequences induced in T cells and/or tumor cells by targeting these immune checkpoint pathways. Laboratory studies provide evidence that inhibitory checkpoint pathways can alter metabolic reprogramming of T cells [15, 230]. T cells receiving PD-1 signals are unable to engage in glycolysis, glutaminolysis, or amino acid metabolism but display an increased rate of FAO [15]. This effect of PD-1 is due to inhibition of glucose and glutamine transport as well as inhibition of HK2, which catalyzes the first step of glycolysis. These studies also determined that PD-1 promotes FAO of endogenous lipids by inhibiting the lipid oxidation PI3K pathway, resulting in increased expression of the rate-limiting enzyme of mitochondrial FAO carnitine palmitoyltransferase 1A (CPT1A), which plays an important role in the utilization of fatty acids as an energy source [231]. In addition, PD-1 induces lipolysis, as determined by the increase of the major triacylglycerol hydrolase desnutrin/adiposite triglyceride lipase (ATGL) and release of fatty acids and glycerol. Concomitantly, PD-1 decreases lipid biosynthesis, which normally occurs during T cell activation, by abrogating the induction of fatty acid synthase (FASN). Consistent with the increased rate of FAO, PD-1 induces a significant elevation of the ketone body 3-hydroxybutyrate, which is produced during FAO. Compared to T cells activated without PD-1 ligation, activated T cells receiving PD-1 signals have lower extracellular acidification rate (ECAR), an indicator of glycolysis, and lower oxygen consumption rate (OCR), an indicator of oxidative phosphorylation, but have higher OCR/ECAR ratio [15]. These findings indicate that in contrast to proliferating T cells, which preferentially use glycolysis for energy production, T cells receiving PD-1 signals are rather metabolically quiescence and preferentially use oxidative phosphorylation over glycolysis as indicated by the higher OCR/ECAR ratio. The enhancement of FAO also points to a mechanistic explanation for the longevity of T cells receiving PD-1 signals in patients with chronic infections and cancer and for their capacity to be reinvigorated by PD-1 blockade. Thus, PD-1 ligation alters the metabolic reprogramming induced upon T cell activation by inhibiting glycolysis and promoting FAO. In contrast, although CTLA-4 inhibits expression of the glutamine and glucose transporters, it inhibits glycolysis without augmenting CPT1A and FAO, suggesting that CTLA-4 maintains immune quiescence by preserving the metabolic profile of non-stimulated cells. The role of PD-1 signaling in restraining T cell glucose metabolism in vivo is also supported by another study in which allogeneic PD-L1^−/−^ bone marrow transplant recipients had elevated levels of Glut1 and lactate production [232].

In addition to its effects on metabolic reprogramming of T cells, the PD-1: PD-L1 pathway may also have implications on the metabolism of cancer cells. It has been observed that subpopulations of established human and murine melanoma cell lines as well as subpopulations of malignant cells in melanomas from patients’ biopsies express PD-1 [233, 234]. Unlike T cells in which PD-1 ligation causes inhibition of PI3K/Akt and MAPK pathways, PD-1 ligation in melanoma cells was found to activate these pathways and to induce mTOR signaling. In this context, ligation of PD-1 with PDL-1 in melanoma cells augments the mTOR-signaling pathway and promotes the expression of glycolytic enzymes, which correlate with tumor growth. Thus, tumor cells may use this pathway not only to escape immune response by suppressing activation and expansion of tumor-infiltrating T cells but, potentially, to actively support their growth by triggering mTOR signaling in trans in neighboring tumor cells thereby creating an intra-tumoral growth signal. It has also been reported that PD-L1 expressed on tumor cells can activate glycolysis and cancer cell growth [16]. Although it is unclear how PD-L1, which has a short cytoplasmic chain without an evident signaling motif, would be ligated under physiologic conditions to trigger tumor-specific activation of glycolytic metabolism, together these findings [16, 234] suggest that the PD-1: PD-L1 pathway may have significant implications not only on T cell anabolic metabolism but also on cancer cell metabolism and growth.

## Harnessing metabolism therapeutically against cancer

So far there has been an extensive characterization of the metabolic features and aberrations in cancer. Cancer cell growth and survival relies on altered metabolic pathways such as aerobic glycolysis, fatty acid synthesis, and glutamine metabolism. In addition, emerging evidence points to a link between resistance in cancer treatment and deregulated cancer metabolism. Targeting cancer metabolism has therefore emerged as a promising new strategy for the development of anticancer agents either used alone or in combination therapies. Such drugs target signaling mediators including enzymes and transcription factors linked to pathways involved in cancer metabolism [235]. To date, only few of these drugs are FDA approved due to unwanted side effects, whereas many are in clinical testing or at pre-clinical stage of development [236, 237]. Examples of such drugs targeting the glycolytic pathway include inhibitors of GLUTs, HK, PKM2 or LDHA [235, 236, 238]. Other drugs target the PPP through inhibition of G6P with the goal to inhibit generation of nucleotide precursors. A G6P inhibitor, 6-aminonictinamide (6-AN), has efficacy against cancer cells in vitro [239, 240]. Other inhibitors of the PPP pathway such as resveratrol and dehydroepiandrosterone (DHEA) have also shown promising in vitro anti-cancer effects [235, 241]. Compounds targeting lipid metabolism have also been tested as anti-cancer drugs with the goal to decrease energy generation through FAO or to limit precursors for synthesis of fatty acid, which are necessary for cancer cell proliferation. For example, metformin, the FDA-approved and widely used anti-diabetic drug inhibits acetyl-CoA carboxylase (ACC) through increased AMPK activation, and there are preclinical and clinical data suggesting its anticancer effects [236, 242–244]. In addition, inhibitors of FASN such as the FDA-approved drug orlistat are under preclinical evaluation for cancer treatment [235, 242]. Furthermore, inhibitors of FAO, such as the CPT1 inhibitor ranolazine, an FDA-approved compound for the treatment of angina, have shown promising anti-cancer effects [235, 242, 245, 246]. Despite the beneficial effects on anti-tumor treatment of such metabolism-targeting drugs, potential effects on the immune system have not yet been examined. However, it should be noted that when used in vivo, such metabolism-targeting drugs might mediate unwanted side effects on healthy tissues and organs that depend on the metabolic pathways targeted. For example, preclinical studies with 6-AN showed severe cytotoxicity on neuronal and hematopoietic cells [239, 240].

After the clinical success of antibodies against checkpoint inhibitors in multiple types of cancer, the role of the adaptive immune system in fighting cancer has become unequivocal. As a consequence, every effort to develop anti-tumor drugs is now accompanied by tests of such drugs on immune cells with the goal to confirm that no unwanted immunosuppressive function that would compromise anti-tumor immunity will be induced. Ideally, anti-tumor drugs or combination therapies should prevent tumor growth but simultaneously favor prolonged anti-tumor T cell function so that functional anti-tumor T cells can develop and possibly synergize with the tumor-specific cytotoxic functions of the therapeutic compound. A major goal of novel immunomodulatory approaches is the generation of tumor-specific Tm in parallel to the generation of Teff cells. This will allow for sustained immune-mediated anti-tumor function instead of a transient anti-tumor effect. For example, metformin, an anti-diabetic drug that, as mentioned above, has also shown direct clinical efficacy in cancer [243, 244], is an AMPK activator. As a consequence, metformin inhibits mTOR and glycolysis, thereby inhibiting tumor growth. Importantly, metformin also inhibits glycolysis by mediating direct inhibitory effects on key components of the glycolytic pathway including the rate-limiting enzyme of glycolysis HK2 [247, 248]. However, via these mechanisms metformin also promotes development of Treg [18] and long-lived Tm cells [154]. In addition, mTOR inhibiting compounds such as rapamycin can also have metabolism-targeting effects on T cells, and although rapamycin is traditionally being used as immunosuppressant, it can promote memory CD8^+^ T cell formation when administered after an acute viral or bacterial infection [81, 154, 155]. Thus the use of metabolism-targeting drugs together with checkpoint inhibitors might alter the activation and differentiation program of tumor-specific T cells and prevent the generation of exhausted T cells.

## Conclusions and future directions

T cells and cancer cells inexorably share metabolic programs and preferences, and thus there is high competition for nutrients between cancer and T cells within the tumor microenvironment. Nutrient deprivation, increased metabolic waste, and the ability of tumors to express inhibitory ligands impair the metabolic fitness and capacity of T cells to uptake and utilize nutrients. Additionally, metabolism determinants of the tumor microenvironment drive T cells to exhaustion and Treg differentiation programs rather than Teff and Tm phenotypes leading to impaired antitumor responses. The changes and adaptations in the tumor microenvironment most likely are not limited to solid tumors because leukemia and lymphoma cells have similar metabolic characteristics with solid tumors and often express immunomodulatory ligands [249–252]. In addition, lymphomas may also contain infiltrating T cells with an exhausted phenotype similar to that identified in chronic viral infections or solid tumors [249]. Thus, drugs that directly target key metabolic enzymes or their upstream regulators will likely interfere with metabolism of both cancer and T cells in which core cell signaling and metabolic pathways converge. Understanding the similarities and differences of metabolic vulnerabilities of T cells and cancer may lead to the development of single-target or combination-based therapies to modify metabolism of the tumor niche thereby targeting both cancer cells and immune cells. Identification of such specific changes in oncometabolites and immunometabolites may define not only novel therapeutic targets but also biomarkers for assessment of therapeutic responses to tumor-immunotherapy combined with metabolism-targeting drugs. The ultimate goal is to design metabolism-based treatment strategies to attack and eradicate cancer while promoting effective and sustainable anti-tumor T cell responses.

## Abbreviations

AMPK: AMP-activated protein kinase
Akt1: RAC-alpha serine/threonine-protein kinase
ATP: adenosine triphosphate
FAO: fatty acid oxidation
CD28: cluster of differentiation 28
CPTI: carnitine palmitoyltransferase 1
ER: endoplasmatic reticulum
FoxP3: forkhead box P3
FOXO: forkhead box subfamily O
GLS: glutaminase
HIF1α: hypoxia-inducible factor 1 α
HK: hexokinase
IDO: indoleamine-pyrrole 2,3-dioxygenase
IL-7: interleukin 7
IL-15: interleukin 15
IL-7R: interleukin 7 receptor
IL-15R: interleukin 15 receptor
mTOR: mammalian target of rapamycin
NADPH: nicotinamide adenine dinucleotide phosphate
NRF2: nuclear factor erythroid 2-related factor 2
OXPHOS: oxidative phosphorylation
PD-1: programmed cell death protein 1
PDL-1/2: PD-1 ligand ½
PFK2: phosphofructokinase 2
PGC1a: peroxisome proliferator-activated receptor gamma coactivator 1-alpha
PI3K: phosphoinositide 3-kinase
PKM2: pyruvate kinase M2
PTEN: phosphatase and tensin homolog
SCO2: synthesis of cytochrome c oxidase 2
SLC1A5: solute carrier family 1 (neutral amino acid transporter), member 5
SRC: spare respiratory capacity
TCA: tricarboxylic acid cycle
TCR: T cell receptor
TIGAR: TP53-induced glycolysis and apoptosis regulator
